# Pimaradienoic Acid Inhibits Carrageenan-Induced Inflammatory Leukocyte Recruitment and Edema in Mice: Inhibition of Oxidative Stress, Nitric Oxide and Cytokine Production

**DOI:** 10.1371/journal.pone.0149656

**Published:** 2016-02-19

**Authors:** Sandra S. Mizokami, Miriam S. N. Hohmann, Larissa Staurengo-Ferrari, Thacyana T. Carvalho, Ana C. Zarpelon, Maria I. Possebon, Anderson R. de Souza, Rodrigo C. S. Veneziani, Nilton S. Arakawa, Rubia Casagrande, Waldiceu A. Verri

**Affiliations:** 1 Departamento de Ciências Patológicas - Centro de Ciências Biológicas, Universidade Estadual de Londrina, Londrina, Paraná, Brazil; 2 Departamento de Ciências Farmacêuticas - Centro de Ciências de Saúde, Universidade Estadual de Londrina, Londrina, Paraná, Brazil; 3 Núcleo de Pesquisa em Ciências Exatas e Tecnológicas, Universidade de Franca, Franca, São Paulo, Brazil; French National Centre for Scientific Research, FRANCE

## Abstract

Pimaradienoic acid (PA; ent-pimara-8(14),15-dien-19-oic acid) is a pimarane diterpene found in plants such as *Vigueira arenaria* Baker (Asteraceae) in the Brazilian savannas. Although there is evidence on the analgesic and *in vitro* inhibition of inflammatory signaling pathways, and paw edema by PA, its anti-inflammatory effect deserves further investigation. Thus, the objective of present study was to investigate the anti-inflammatory effect of PA in carrageenan-induced peritoneal and paw inflammation in mice. Firstly, we assessed the effect of PA in carrageenan-induced leukocyte recruitment in the peritoneal cavity and paw edema and myeloperoxidase activity. Next, we investigated the mechanisms involved in the anti-inflammatory effect of PA. The effect of PA on carrageenan-induced oxidative stress in the paw skin and peritoneal cavity was assessed. We also tested the effect of PA on nitric oxide, superoxide anion, and inflammatory cytokine production in the peritoneal cavity. PA inhibited carrageenan-induced recruitment of total leukocytes and neutrophils to the peritoneal cavity in a dose-dependent manner. PA also inhibited carrageenan-induced paw edema and myeloperoxidase activity in the paw skin. The anti-inflammatory mechanism of PA depended on maintaining paw skin antioxidant activity as observed by the levels of reduced glutathione, ability to scavenge the ABTS cation and reduce iron as well as by the inhibition of superoxide anion and nitric oxide production in the peritoneal cavity. Furthermore, PA inhibited carrageenan-induced peritoneal production of inflammatory cytokines TNF-α and IL-1β. PA presents prominent anti-inflammatory effect in carrageenan-induced inflammation by reducing oxidative stress, nitric oxide, and cytokine production. Therefore, it seems to be a promising anti-inflammatory molecule that merits further investigation.

## Introduction

Inflammation is a common mechanism of many diseases. Despite the importance of controlling inflammation, the current anti-inflammatory drugs present many side effects that limit their clinical use [1]. Therefore, it is still necessary to develop novel anti-inflammatories. Inflammatory cardinal signs include the development of pain, erythema, heat, edema, and loss of function. An important non-clinical sign of inflammation involves the recruitment of leukocytes to the inflammatory foci [2]. Upon an inflammatory stimulus, resident cells produce cytokines to communicate the threat to other cells and respond to it. Cytokines activate the endothelial cells to express adhesion molecules and chemoattract leukocytes to the inflammatory foci [3]. These leukocytes are mainly neutrophils in acute inflammation. At the inflammatory foci, neutrophils produce reactive oxygen species such as superoxide anion and nitric oxide [3], which induce tissue damage by oxidative stress and forming the highly reactive and deleterious peroxynitrite [4]. Due to the harmful effects of exacerbated inflammation, the use of anti-inflammatories is a useful clinical tool to control inflammation and reduce tissue damage [1].

*ent*-pimara-8(14),15-di*en*-19-oic acid is a pimarane diterpene known as pimaradienoic acid (PA). Various plants produce PA, especially *Vigueira arenaria* Baker (Asteraceae), which presents PA in high concentrations. *V*. *arenaria* is a herbaceous plant native of the Brazilian savannas [5–7]. The pharmacological activities of PA include the antispasmodic and relaxant actions on vascular smooth muscle and inhibition of rat carotid contractions [7–9], and antimicrobial activity [10, 11].

Furthermore, evidence supports the anti-inflammatory action of PA as follows. *In vitro*, PA inhibits LPS-induced production of IL-6, nitric oxide (NO) and prostaglandin (PG) E_2_ as well as cyclooxygenase (COX)-2 and inducible nitric oxide synthase (iNOS) mRNA expression, and NF-кB activation in RAW 264.7 macrophages [12]. *In vivo*, PA inhibited carrageenan-induced inflammatory paw edema in mice [13]. Moreover, PA inhibited acetic acid-induced inflammatory abdominal writhing response [14, 15]. PA also inhibited carrageenan- and complete Freund´s adjuvant-induced mechanical hyperalgesia by inhibiting paw skin NFκB activation, cytokine production, and activating the nitric oxide (NO)/ cyclic guanosine monophosphate (cGMP)/ ATP-sensitive potassium channels signaling pathway [14]. Importantly, PA does not induce gastric or liver damage within a seven days treatment protocol [14], which corroborates its potential applicability to control inflammation with reduced side effects compared to non-steroidal anti-inflammatory drugs. It is also not expected that PA would present corticoid-like side effects since these occur as a result of binding to corticoid receptor and activation of corticoid responsive genes [16].

Despite the above-mentioned evidence, it remains to be determined whether PA inhibits other inflammatory parameters in addition to paw edema and its mechanisms *in vivo*. Therefore, the anti-inflammatory effect and mechanisms of PA were investigated in the present study.

## Materials and Methods

### Animals

Male Swiss mice (20–25 g), from Universidade Estadual de Londrina, Londrina, Paraná, Brazil, were used in this study. Mice housing was in standard clear plastic cages with free access to food and water, a light/dark cycle of 12:12 h, and kept at 21°C. In all behavioral testing, experiments were performed between 9 a.m. and 5 p.m. in a temperature-controlled room. Before sample collection, mice were terminally anesthetized with 3% isoflurane followed by decapitation. The Ethics Committee of the Universidade Estadual de Londrina (CEUA-UEL) specifically approved this study (process number 1531.2013.76). Every effort was made to minimize the number of animals used and their suffering.

### Plant material

M. Magenta collected *Viguiera arenaria* Baker (Asteraceae) at Itirapina—SP (22°13 S, 47°54 W, SP, Brazil), identified the plant material, and deposited a voucher specimen under the code SPF #61 in the herbarium of the University of São Paulo (SP, Brazil). Prof. F. B. Costa gently provided the plant material [7, 8, 14]. The Genetic Heritage Management Council (CNPq, Brazil, Process #010055/2012-6) authorized collecting *V*. *arenaria*. It is noteworthy to mention that *V*. *arenaria* is not endangered or protected specie.

### Extraction and isolation

Extraction of air-dried tuberous roots (980 g) from *V*. *arenaria* was with CH_2_Cl_2_ for 30 minutes using a sonicator to yield 82 g of crude extract. After suspension in MeOHyH_2_O (9:1, v/v), the crude extract was exhaustively washed with hexane and CH_2_Cl_2_ to yield 39.5 g (hexane phase) and 25.0 g (CH_2_Cl_2_ phase). The hexane phase was chromatographed over Si gel using vacuum liquid chromatography to yield six fractions: F1 (0.5 g), F2 (13.3 g), F3 (14.2 g), F4 (5.3 g), F5 (2.4 g) and F6 (3.6 g). Fraction F2 furnished the diterpene PA. Isolation and purification steps were carried out by flash chromatography (hexane-EtOAc), PTLC (Si gel, hexane-EtOAc or hexane-CHCl_3_) and recrystallization from MeOH. The structure of the diterpene was established by comparison of the 1H and 13C NMR spectral data with those reported in the literature [6, 7, 14].

### Drugs

The compounds used in this study were carrageenan and dimethyl sulfoxide (DMSO) (Santa Cruz Biotechnology, Santa Cruz, CA, United States). Nitroblue Tetrazolium (NBT) (Sigma-Aldrich, St. Louis, MO, USA). Mouse IL-1β and TNF-α ELISA kits obtained from eBioscience (San Diego, CA, USA).

### Experimental protocols

The optical rotation of PA was measured in CHCl_3_ using a Perkin Elmer 241 polarimeter. Nuclear magnetic resonance (NMR) spectra were run on a Bruker DPX 400 spectrometer (400 MHz for 1H and 100 MHz for 13C). Samples were dissolved in CDCl_3_, and the spectra were calibrated with the solvent signals at δ 7.26 (1H) and δ 77.0 (13C). Mass spectrometric analysis was performed at low resolution on a Micromass Quattro-LC instrument (Manchester, UK) provided with an ESI ion source and a triple quadrupole mass analyzer. Solutions were dissolved in MeOH-H_2_O 8:2 (v/v) and infused into the ESI source at a flow-rate of 5 μL/minute, using a Harvard apparatus model 1746 (Holliston, MA) syringe pump. Vaccum-liquid chromatography (VLC) was carried out using silica gel 60H (Merck, art. 7736) in glass columns with 5–10 cm i.d.29 High-performance liquid chromatography (HPLC) analysis was accomplished using a Shimadzu CBM-20A liquid chromatography controller, operating with LC solution software, equipped with a Shimadzu UV-DAD detector SPD-M20A and a Shimadzu ODS column (4.6 x 250 mm, 5 μm, 100 Å) [6, 7].

During the experiments, mice received per oral (p.o.) treatment with PA (0.1, 1 and 10 mg/kg) or vehicle (saline) 30 minutes before intraperitoneal (i.p.; 500 μg) or intraplantar (i.pl.; 300 μg) carrageenan injection. The doses of the inflammatory stimulus were determined previously in our laboratory in pilot studies and previous work [17–19]. The number of total leukocytes, neutrophils, and mononuclear cells recruited to the peritoneal cavity was evaluated 6 hours after carrageenan (500 μg/cavity) injection. Paw edema was evaluated 0–5 hours after carrageenan (300 μg/paw) injection, and at 5 hours animals were euthanized and paw skin samples were collected for MPO activity. Oxidative stress in the paw, and superoxide anion levels, quantification of NBT positive cells, and nitric oxide production in the peritoneal cavity were assessed at 3 hours following carrageenan-stimuli. Further, and TNFα and IL-1β cytokines levels in the peritoneal cavity were determined at 6 hours. The inflammatory models and time points of sample collection were of pilot studies and previous work [18, 20–23].

### Recruitment of leukocyte to peritoneal cavity in mice

The recruitment of leukocytes to peritoneal cavity was assessed 6 hours after carrageenan injection. After mice euthanasia, the peritoneal cavity cells were harvested by washing the cavity with 2 mL of phosphate-buffered saline (PBS) containing 1 mM of EDTA. The volumes recovered were similar in all experimental groups and equated to approximately 90% of the injected volume. The total leukocytes counts were performed with a Newbauer chamber, and differential cell counts (100 cells total) were carried out on cytocentrifuge slides (Cytospin 3; Shandon Southern Products, Astmoore, UK) stained with panotic solutions (Laborclin, Pinhais, Paraná, Brazil). The results are the number of total leukocytes, neutrophils or mononuclear cells x 10^6^ [17, 18, 24].

### Paw edema test

The volume of the mice paw was measured using an analog caliper (Digmatic Caliper, Mitutoyo Corporation, Kanagawa, Japan) before (basal) and at indicated time points after carrageenan injection (VT). The amount of paw swelling for each mouse and the difference between VT and basal was the edema value (mm/paw)[18, 25].

### Myeloperoxidase activity (MPO)

The neutrophil migration to paw was indirectly evaluated by the MPO activity kinetic-colorimetric assay [26]. Briefly, samples were collected in 50 mM K_2_PO_4_ buffer (pH 6.0) containing 0.5% HTAB and were homogenized using Ultra-Turrax^®^ (IKA T10 Basic, CQA Química, Paulínea, SP). Then the homogenates were centrifuged at 16,100 *g* for 2 minutes at 4°C. Fifteen μL of the resulting supernatant was mixed with 200 μL of 50 mM phosphate buffer, pH 6.0, containing 0.167 mg/mL o-dianisidine dihydrochloride and 0.05% hydrogen peroxide and was assayed spectrophotometrically for MPO activity determination at 450 nm (BEL SP2000UV, Photonics, São Paulo, SP, Brazil). The results of MPO activity were expressed as the number of neutrophils per mg of tissue by using a standard curve of neutrophils (100,000–1,562.5 cells). Neutrophils for the standard curve were from the peritoneal cavity of Swiss mice, 6 hours after i.p. stimulus with thioglycolate broth (1 mL, 5%, Becton Dickinson, MD, USA). The number of neutrophils was determined by total counts in Neubauer chamber and differential counts in slides stained by Rosenfelt method. We obtained 96% of neutrophils in a pool of 10 mice. Neutrophils were suspended in K_2_HPO_4_ buffer containing HTAB and stored at -80°C until use.

### Reduced glutathione (GSH) levels measurement

Samples of paw skin were collected and maintained at −80°C for at least 48 h. Samples were homogenized in 200 μL of 0.02 M EDTA. The homogenate was mixed with 25 μL of trichloroacetic acid 50% and was homogenized three times over 15 minutes. The mixture was centrifuged (15 minutes x 1500 *g* at 4°C). The supernatant was added to 200 μL of 0.2 M TRIS buffer, pH 8.2, and 10 μL of 0.01M DTNB. After 5 minutes, the absorbance was measured at 412 nm (Multiskan GO, Thermo Scientific) against a blank reagent with no supernatant. A standard GSH curve was formed. The results are GSH per mg of protein.

### ABTS and FRAP assays

The ability of samples to resist oxidative damage was determined by their free radical scavenging (ABTS [2,2'-Azinobis-3-ethylbenzothiazoline 6-sulfonic acid] assay) and ferric reducing ability (FRAP assay). The tests were adapted to a 96-well microplate format as previously described [21]. Plantar tissue samples were collected 3 hours after carrageenan i.pl. injection (300 μg, 25 μL) and homogenized immediately in ice-cold KCl buffer (500 μL, 1.15% w/v). The homogenates were centrifuged (200 *g* × 10 minutes at 4°C), and the supernatants were used in both assays. Diluted ABTS solution (200 μL) was mixed with 10 μL of sample in each well. After 6 minutes of incubation at 25°C, the absorbance was measured at 730 nm. For FRAP assay, the supernatants (10 μL) were mixed with the freshly prepared FRAP reagent (150 μL). The reaction mixture was incubated at 37°C for 30 minutes, and the absorbance was measured at 595 nm (Multiskan GO Thermo Scientific). The results of ABTS and FRAP assays were equated against a standard Trolox curve (0.02–20 nmol).

### NBT assay

The quantification of superoxide anion production in tissue homogenates (10 mg/mL in 1.15% KCl) was performed using the NBT assay as previously described [27]. Briefly, 50 μL of homogenate were incubated with 100 μL of NBT (1 mg/mL) in 96-well plates at 37°C for 1 hour. The mixture was then carefully removed from wells and the reduced formazan solubilized by adding 120 μL of KOH 2 M and 140 μL of DMSO. The absorbance was measured at 600 nm (Multiskan GO ThermoScientific). The weight of samples was used for data normalization and results presented as NBT reduction (OD at 600 nm).

### Nitric oxide production

Nitrite (NO_2_^-^) concentration in peritoneal exudate was determined by the Griess reaction as an indicator of nitric oxide production as previously reported [28]. Briefly, 100 μL of samples and 100 μL of Griess reagent (mix of 2% sulphanilamide in 5% phosphoric acid and 0.2% N-(1-naphthyl) ethylenediamine hydrochloride—NEED) were mixed in 96-well ELISA plate. Absorbance was measured at 550 nm, and the levels of NO_2_^-^ were determined using a standard curve of NaNO_2_. Results are μM of NO_2_^-^ per cavity.

### Cytokine measurement

Mice were treated with vehicle or PA (10 mg/kg, p.o.) 30 minutes before carrageenan (500 μg/cavity) stimulus. Six hours after the carrageenan injection, animals were terminally anesthetized, and the peritoneal cavity cells were harvested and washing by introducing 1 mL PBS containing 1 mM of EDTA. The cytokines (TNF-α and IL-1β) levels were determined by an enzyme-linked immunosorbent assay (ELISA) according to manufacturer’s instructions (eBioscience) and as described previously [23]. Results are pg of cytokine/cavity.

### Statistical analysis

Results are presented as means ± SEM of measurements made on 6 mice in each group per experiment and are representative of two independent experiments. Differences between groups were evaluated by analysis of variance (ANOVA) followed by Tukey’s test. All statistical analyzes were performed using Graph Pad Prism (La Jolla, 5 CA). Statistical differences were considered to be significant at *P*<0.05.

## Results

### Pimaradienoic acid (PA) inhibits carrageenan-induced total leukocyte and neutrophil recruitment in the peritoneal cavity

**Fig 1:**
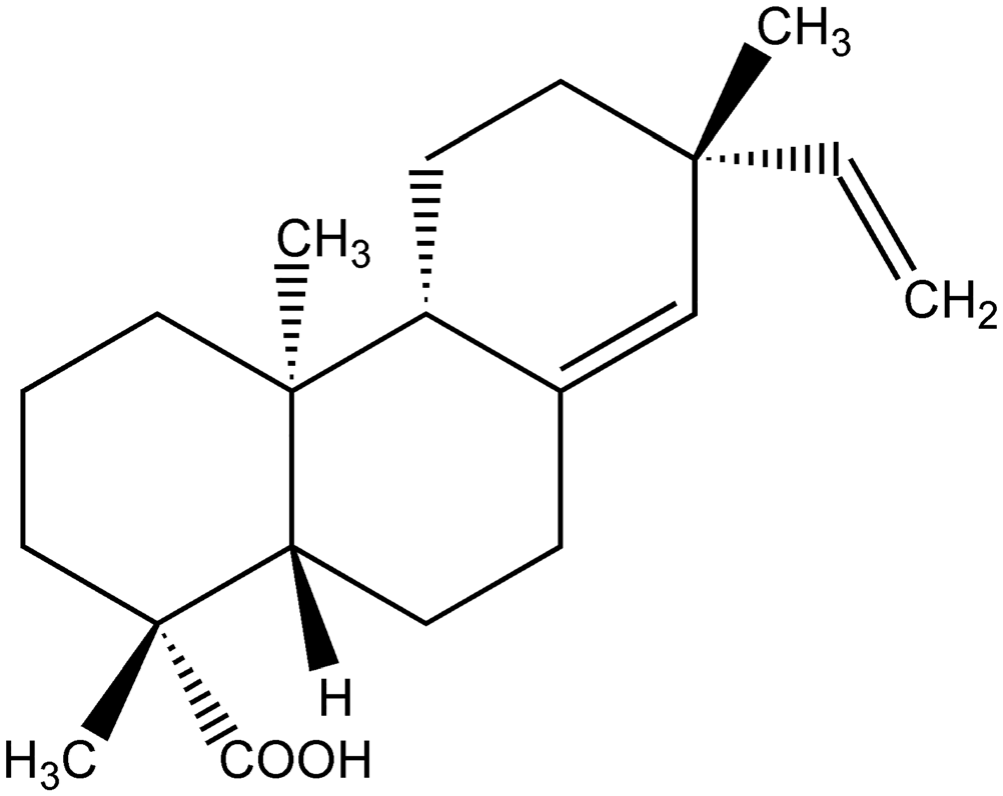
Pimaradienoic acid (PA) structure. Chemical structure of PA[29].

**Fig 2:**
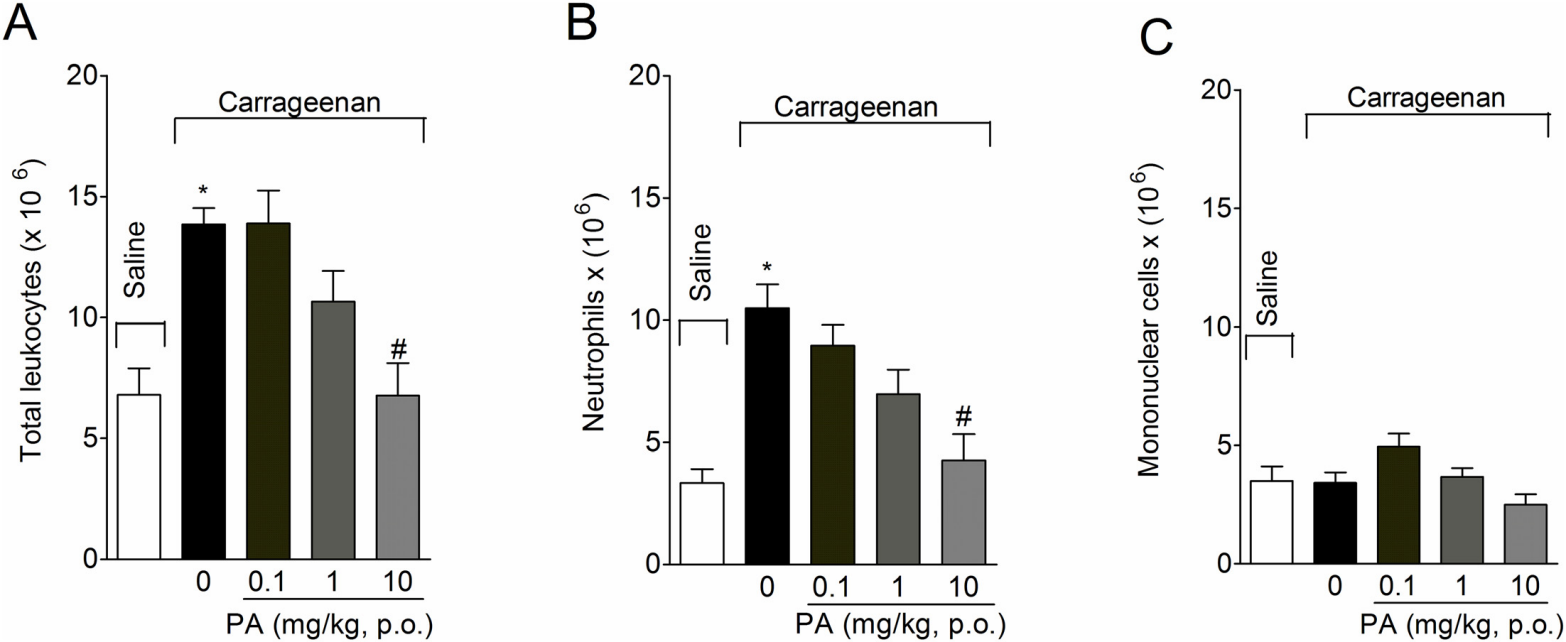
Pimaradienoic acid (PA) inhibits carrageenan-induced total leukocyte and neutrophil recruitment in the peritoneal cavity. Mice were treated per oral (p.o.) with PA (0.1–10 mg/kg) or vehicle (DMSO 2% diluted in saline) 30 minutes before carrageenan (500 μg/ cavity) intraperitoneal (i.p.) injection. The (A) total number of leukocytes, (B) neutrophils and (C) mononuclear cells was evaluated 6 hours after carrageenan injection. Results are means ± SEM of six mice per group per experiment and are representative of two separate experiments. [*p < 0.05 compared to the saline group; #p < 0.05 compared to the vehicle group (One-way ANOVA followed by Tukey’s test)].

Mice received per oral (p.o.) treatment with PA (Fig 1)[29] (0.1–10 mg/kg, 2% DMSO diluted in saline) 30 minutes before intraperitoneal (i.p.) injection of carrageenan (500 μg/cavity) and the recruitment of total leukocytes, neutrophils, and mononuclear cells was assessed at 6 hours after stimulus. Carrageenan injection induced a significant increase in the recruitment of total leukocytes (Fig 2A) and neutrophils (Fig 2B), but not mononuclear cells (Fig 2C). Only the dose of 10 mg/kg of PA inhibited carrageenan-induced total leukocyte (Fig 2A) and neutrophil (Fig 2B) recruitment. Therefore, 10 mg/kg was selected for the next experiments. No effect was observed in the number of mononuclear cells for any of the doses of PA tested (Fig 2C).

### PA inhibits carrageenan-induced paw edema and myeloperoxidase activity

**Fig 3:**
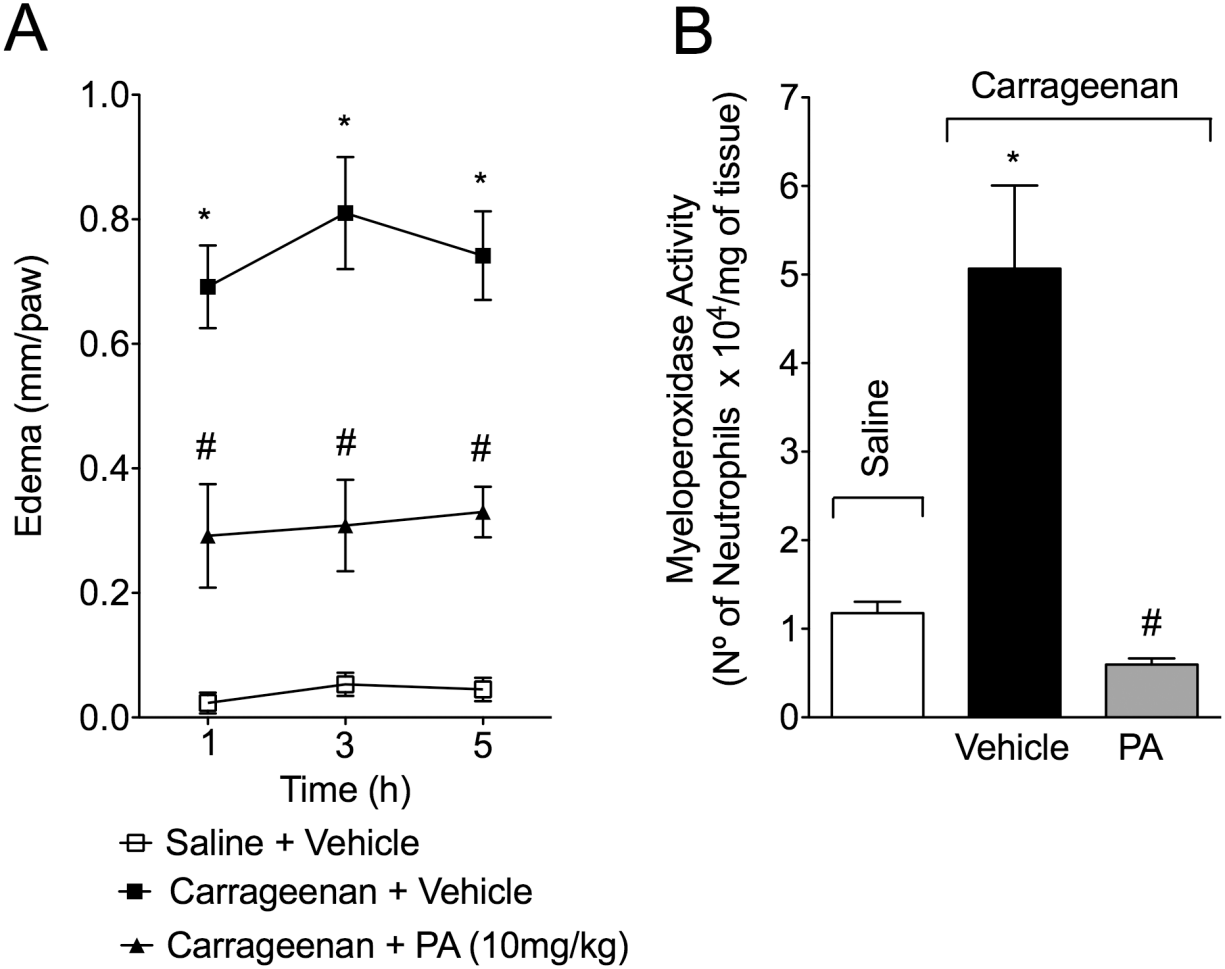
Pimaradienoic acid (PA) inhibits carrageenan-induced paw edema and myeloperoxidase (MPO) activity. Mice were treated per oral (p.o.) with PA (10 mg/kg) or vehicle (DMSO 2% diluted in saline) 30 minutes before the intraplantar (i.pl.) injection of carrageenan (300 μg/paw). The evaluation of (A) paw edema was at 1–5 hours and (B) MPO activity at 5 hours after carrageenan injection. Results are means ± SEM of six mice per group per experiment, and are representative of two separate experiments. [*p< 0.05 compared with the saline group, #p <0.05 compared to the vehicle group (One-way ANOVA followed by Tukey’s test)].

Mice received p.o. treatment with PA (10 mg/kg, 2% DMSO diluted in saline) 30 minutes before intraplantar (i.pl.) injection of carrageenan (300 μg/paw). PA significantly inhibited carrageenan-induced paw edema at 1, 3 and 5 hours (Fig 3A) and myeloperoxidase activity at 5 hours (Fig 3B) after stimulus injection.

### PA inhibits carrageenan-induced oxidative stress in the paw skin

**Fig 4:**
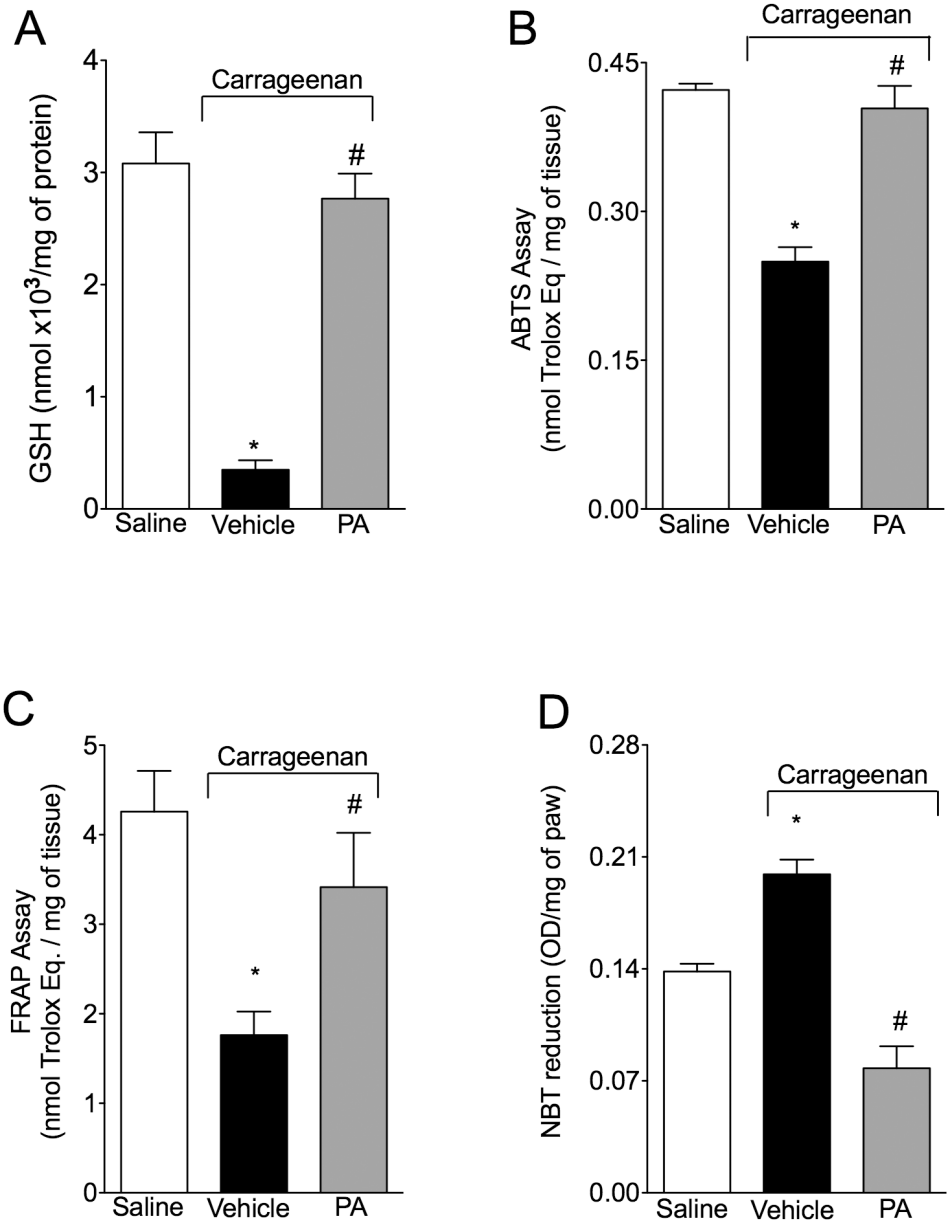
Pimaradienoic acid (PA) inhibits carrageenan-induced oxidative stress. Mice were treated per oral (p.o.) with PA (10 mg/kg) or vehicle (DMSO 2% diluted in saline) 30 minutes before the intraplantar (i.pl.) injection of carrageenan (300 μg/paw). Paw skin (A) GSH levels, (B) ABTS^+^ scavenging activity, (C) the ability to reduce iron (FRAP), and (D) superoxide anion production were determined 3 hours after carrageenan injection. Results are means ± SEM of six mice per group per experiment, and are representative of two separate experiments. [*p<0.05 compared to the saline group; #p<0.05 compared to inflammatory stimulus group. (One-way ANOVA followed by Tukey’s test)].

To assess the protective role of PA on carrageenan-induced oxidative stress; mice received p.o. treatment with PA (10 mg/kg, 2% DMSO diluted in saline) 30 minutes before i.pl. injection of carrageenan (300 μg/paw) and were terminally anesthetized 3 hours after the stimulus. Carrageenan-induced a reduction in GSH levels (Fig 4A), ABTS scavenging ability (Fig 4B) and ferric reducing ability (Fig 4C), which were prevented by PA treatment. In agreement, treatment with PA also inhibited the carrageenan induced increase of superoxide production (NBT assay; Fig 4D). Together, these data demonstrate that PA inhibits carrageenan-induced tissue oxidative stress.

### PA inhibits carrageenan-induced oxidative stress in the peritoneal cavity

**Fig 5:**
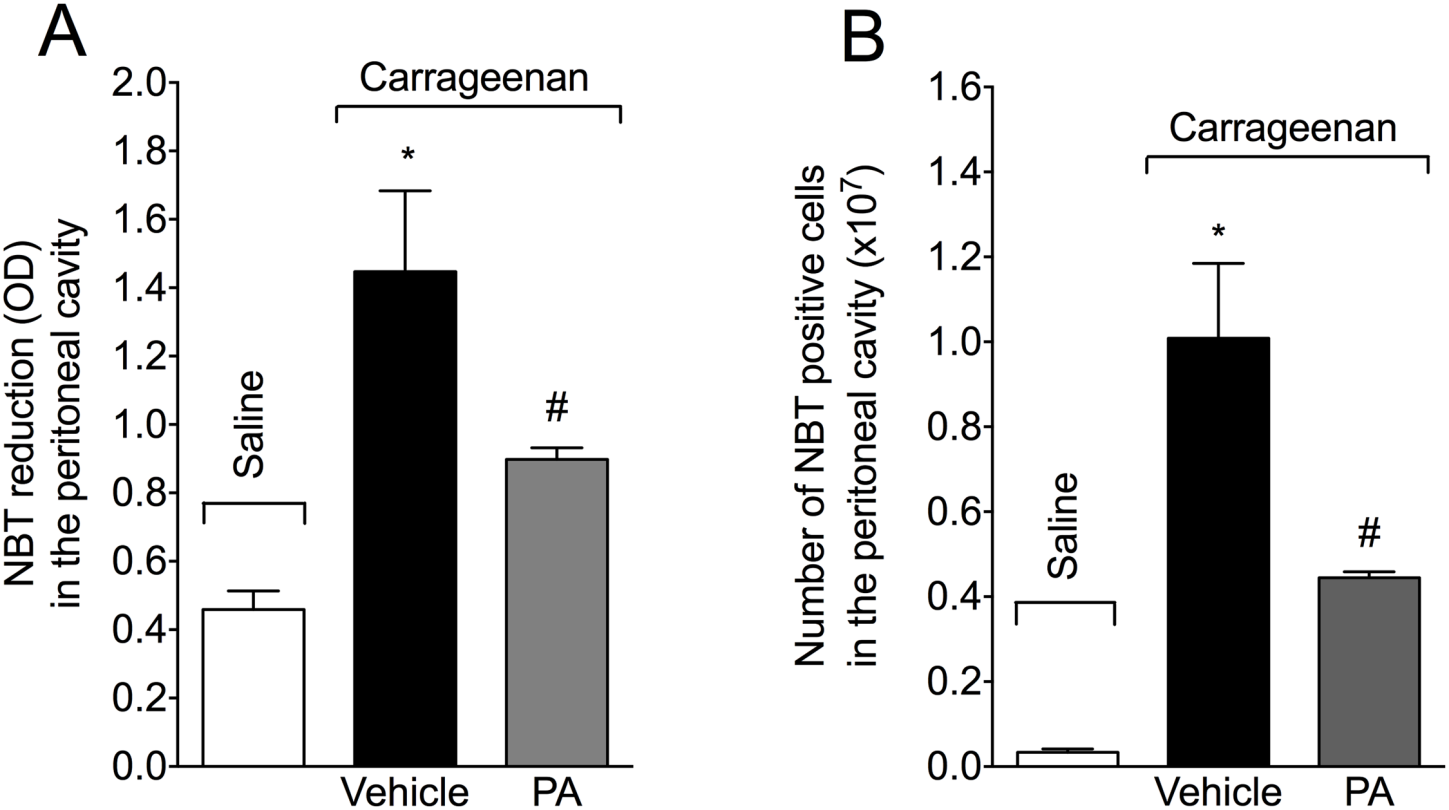
Pimaradienoic acid (PA) inhibited carrageenan-induced superoxide anion production. Mice were treated per oral (p.o.) with PA (10 mg/kg) or vehicle (DMSO 2% diluted in saline) 30 minutes before the intraperitoneal (i.p.) injection of carrageenan (500 μg/cavity). (A) Superoxide anion production and (B) NBT reaction positive cells (presence of formazan crystals) were determined in peritoneal cavity exudates 3 hours after carrageenan injection. Results are means ± SEM of six mice per group per experiment, and are representative of two separate experiments. [*p< 0.05 compared with the saline group, and #p< 0.05 compared to the vehicle group (One-way ANOVA followed by Tukey’s test)].

Mice received p.o. treatment with PA (10 mg/kg, 2% DMSO diluted in saline) 30 minutes before i.p. injection of carrageenan (500 μg/paw). PA significantly inhibited carrageenan-induced NBT reduction in the peritoneal exudate (Fig 5A) at 3 hours, demonstrating that PA inhibits carrageenan-induced superoxide anion production. Furthermore, PA also reduced the number of NBT positive cells in the peritoneal cavity (Fig 5B) at this same time point. Therefore, PA not only reduces the total superoxide anion production (Figs 4D and 5A) but also reduces the number of cells producing superoxide anion (Fig 5B).

### PA inhibits carrageenan-induced nitric oxide (NO) production in the peritoneal cavity

**Fig 6:**
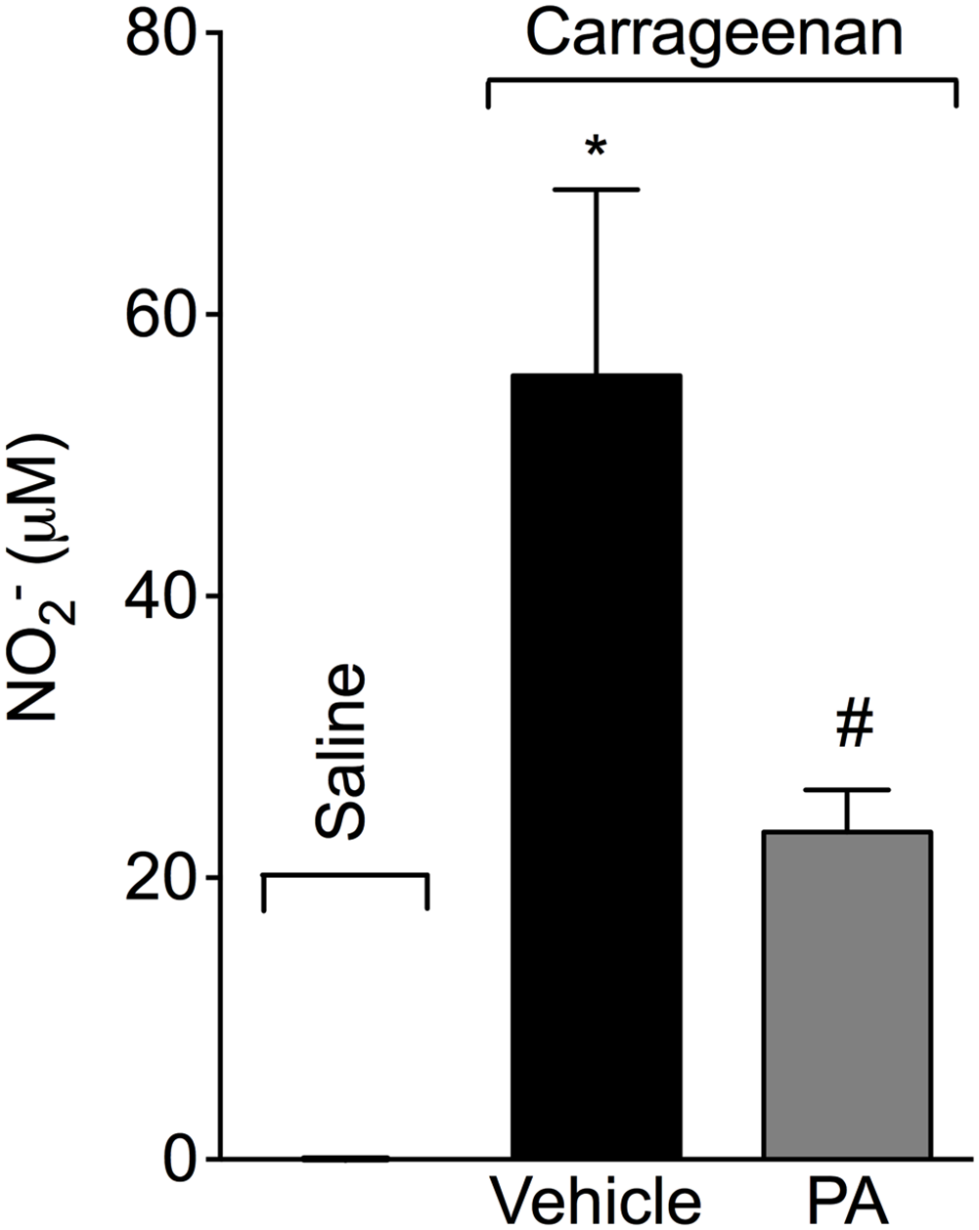
Pimaradienoic acid (PA) inhibits carrageenan-induced nitric oxide (NO) production. Mice were treated per oral (p.o.) with PA (10 mg/kg) or vehicle (DMSO 2% diluted in saline) 30 minutes before the intraperitoneal (i.p.) injection of carrageenan (300 μg/paw). Nitrite production in peritoneal exudates was determined 3 hours after carrageenan injection. Results are means ± SEM of six mice per group per experiment, and are representative of two separate experiments. [*p<0.05 compared with the saline group, and #p< 0.05 compared to the vehicle group (One-way ANOVA followed by Tukey’s test)].

Mice received p.o. treatment with PA (10 mg/kg, 2% DMSO diluted in saline) 30 minutes before i.p. injection of carrageenan (500 μg/paw). PA significantly inhibited carrageenan-induced nitrite production in the peritoneal exudates (Fig 6) at 3 hours.

### PA inhibits carrageenan-induced TNF-α and IL-1β production in the peritoneal cavity

**Fig 7:**
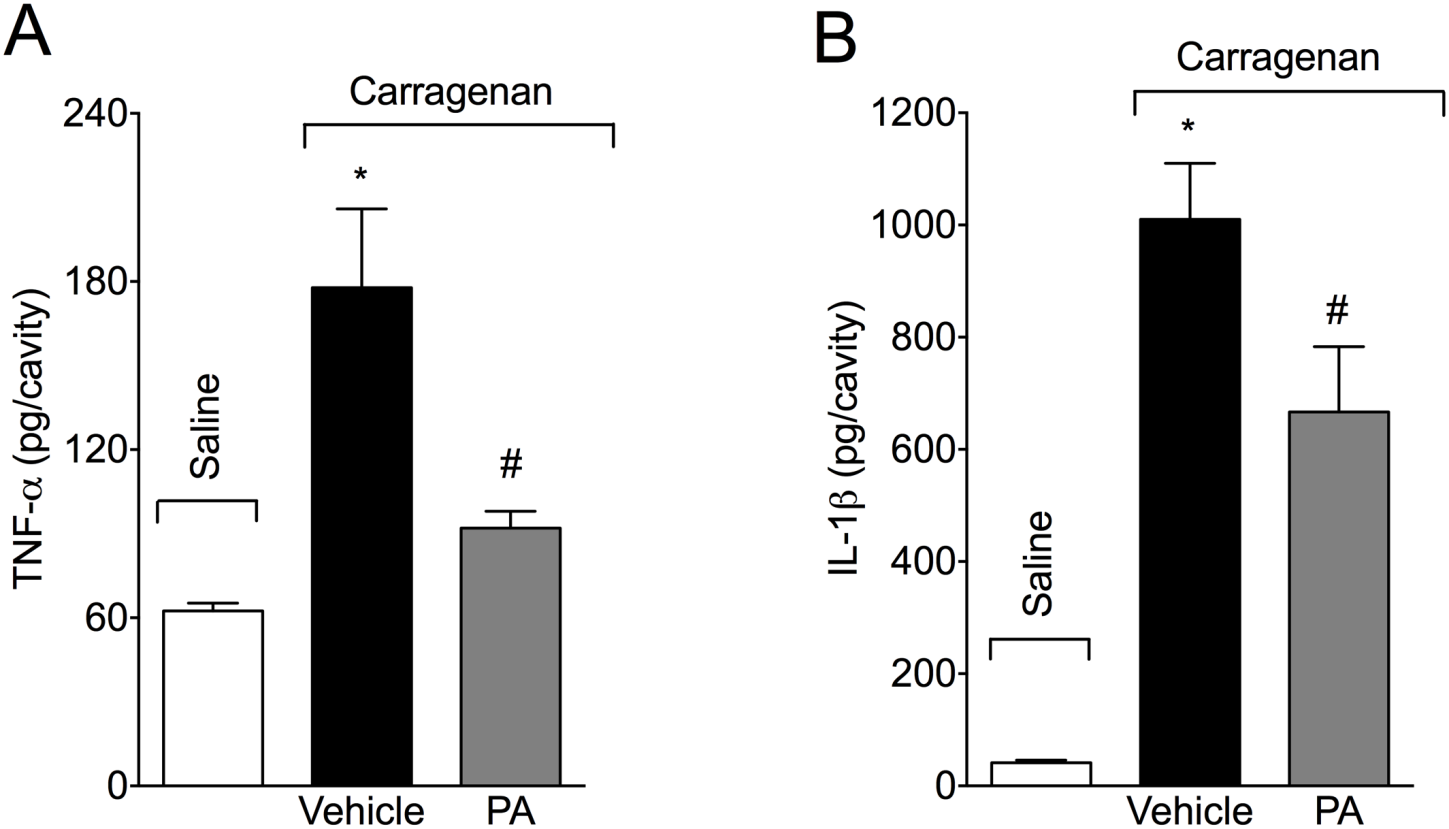
Pimaradienoic acid (PA) inhibits carrageenan-induced peritoneal cytokine production. Mice were treated per oral (p.o.) with PA (10 mg/kg) or vehicle (DMSO 2% diluted in saline) 30 minutes before the intraperitoneal (i.p.) injection of carrageenan (500 μg/cavity). Peritoneal exudate samples were harvested 6 hours after carrageenan injection for (A) TNF-α and (B) IL-1β determination by ELISA. Results are means ± SEM of six mice per group per experiment, and are representative of two separate experiments. [*p<0.05 compared with the saline group, #p<0.05 compared to vehicle group (One-way ANOVA followed by Tukey’s test)].

Mice received p.o. treatment with PA (10 mg/kg, 2% DMSO diluted in saline) 30 minutes before i.p. injection of carrageenan (500 μg/paw). PA significantly inhibited carrageenan-induced TNF-α (Fig 7A) and IL-1β (Fig 7B) production in the peritoneal exudate at 6 hours. Therefore, in addition to inhibiting carrageenan-induced oxidative stress, PA also inhibits carrageenan-induced cytokine production.

## Discussion

The present data demonstrate that pimaradienoic acid (PA) inhibits carrageenan-induced leukocyte recruitment and edema. The mechanisms involved in the anti-inflammatory effect of PA depended on inhibiting oxidative stress, nitric oxide and cytokine production in mice. Importantly, to our knowledge, this is the first study demonstrating that PA reduces oxidative stress *in vivo*.

We have demonstrated that PA inhibits acetic acid-induced writhing response irrespective of the route of administration, i.p., s.c. or p.o. route. Nevertheless, the p.o. route achieved only slightly lower effect than i.p. route, suggesting that the study with PA p.o. is suitable considering that this is a useful and easy route of administration to conscious patients [14]. PA inhibited carrageenan-induced total leukocyte and neutrophil recruitment to the peritoneal cavity in a dose-dependent manner. Neutrophils are the first cell type recruited in innate immune response as observed in the carrageenan model. Therefore, it is coherent that the increase of total leukocytes is predominantly reflecting the neutrophil counts [3]. The dose of PA of 10 mg/kg is the same used in a previous study on the analgesic effect of PA, which suggests that PA at this dose inhibits inflammation and pain. Nevertheless, studies of other groups observed analgesic and anti-inflammatory effects of PA with higher doses than the present study. For instance, PA inhibited carrageenan-induced paw edema by 22% at the dose of 100 mg/kg [13]. We observed up to 64% inhibition of carrageenan-induced paw edema by PA at 3 hours. Possible explanations for this divergence include that PA may present a U or bell shape effect, different doses of carrageenan (50 μg/paw versus 300 μg/paw), mouse strain (ICR versus Swiss mice), method of paw edema evaluation (plethysmometer versus caliper), and time point of evaluation (5 hours versus 1–5 hours) used in [13] and the present study, respectively.

Importantly, the carrageenan-induced paw inflammation was also useful to demonstrate that PA inhibits the increase of myeloperoxidase activity in the paw skin, which is an indirect measurement of neutrophil and macrophage counts. The result on myeloperoxidase in the paw skin corroborated the data on total leukocyte and neutrophil counts in the peritoneal cavity, demonstrating that PA inhibits carrageenan-induced leukocyte recruitment irrespectively of the tissue or cavity. Both neutrophils and macrophages contribute to MPO activity in inflamed tissues, although neutrophils are the major source of MPO, accounting for approximately 5% of their dry mass [26]. Moreover, not all subpopulations of macrophages are MPO sources, and they depend on the type of inflammatory model (acute versus late model) [30]. Furthermore, the present assay did not detect MPO activity of up to 1 x 10^5^ macrophages (data not shown). Taking into account that the carrageenan model is an acute inflammatory model, PA did not alter monocyte recruitment to the peritoneal cavity, and the employed method to assess MPO activity is selective to neutrophils [31–33], we suggest that PA reduces carrageenan-induced inflammation by inhibiting neutrophil recruitment.

Although other mechanisms, for example, apoptosis or macrophage efferocytosis, are also important in the reduction in the number of neutrophils at the site of inflammation [34], this does not seem to be the case for PA. Firstly, apoptotic leukocytes or apoptotic bodies were not observed during the examination of the recruitment of leukocytes to the peritoneal cavity (data not shown). Further, the only evidence of PA-induced apoptosis is DNA damage in liver cells of Swiss mice induced by doses 4–8 fold higher (40–80 mg/kg, p.o.) than used in the present study (0.1–10 mg/kg, p.o.) [35]. Secondly, previous studies have shown the importance of IL-10 in maintaining phagocytosis/efferocytosis during inflammatory conditions [36]. However, PA does not increase IL-10 levels in the paw tissue following carrageenan stimulus but reduces its levels [14]. Collectively, these data suggest that the reduction in the carrageenan-induced increase in the number of neutrophils in the paw and peritoneal cavity of mice is due to the reduction in neutrophil recruitment rather than increasing the neutrophil apoptosis or efferocytosis.

Carrageenan-induced inflammation depends on tissue oxidative stress [18]. As PA inhibited carrageenan inflammation, it was conceivable to determine whether PA also inhibits oxidative stress. Carrageenan significantly reduced GSH levels, the tissue ability to scavenge ABTS cation radical and to reduce iron (FRAP) as well as increased superoxide anion production. PA inhibited all these parameters of oxidative stress in the paw skin. These data on paw skin oxidative stress suggests that the inhibition of carrageenan-induced oxidative stress may also explain the analgesic effect of PA [14]. Only high concentrations (IC_50_ > 100 μg/mL) of PA exhibit DPPH radical scavenging activity *in vitro* [37]. The fact that scavenging free radical is not the unique mechanism that an antioxidant molecule may present might explain this apparent divergence. Inhibition of lipid peroxidation, iron chelation, and scavenging other free radicals are important antioxidant mechanisms [38]. It is noteworthy that the *in vivo* antioxidant effect of PA may be indirect by inhibiting the inflammatory process [39, 40]. Activated neutrophils are major sources of reactive oxygen species (ROS) at the site of inflammation, thus, the reduction in oxidative stress may be the result of reduced neutrophil recruitment by PA [41]. Further, the inhibition of TNF-α and IL-1β by PA may also have contributed to the reduction in oxidative stress since inflammatory cytokines can enhance ROS and reactive nitrogen species (RNS) production via NADPH oxidase and iNOS enzymes, respectively [40, 42].

PA also inhibited carrageenan-induced superoxide anion production in the peritoneal exudate and the counts of NBT positive peritoneal cells. Therefore, the PA inhibition of carrageenan-induced superoxide anion was also accompanied by a reduction of cellular activation of superoxide anion production. Although a pimaradiene diterpene with a similar structure to PA, acanthoic acid, had more prominent effect in reducing ROS production by monocytes/macrophages than by neutrophils [43], a noticeable difference between the amount of ROS induced by the stimuli used to activate monocytes/macrophages and neutrophils could be observed. In this sense, it was difficult to conclude that acanthoic acid acts similarly or differently on the production of ROS by these cells. Thus, it is possible that PA acts on the reduction of ROS production in both macrophages and neutrophils. However, future studies are necessary to elucidate the effects of PA on ROS/RNS production by each cell type.

PA also inhibited carrageenan-induced NO production in the peritoneal exudate. *In vitro* evidence showed that PA inhibits LPS-induced iNOS mRNA expression and NO production by RAW 264.7 macrophages [12]. Macrophages are important cells in the peritoneal cavity and a possible source of NO during inflammation, therefore, a potential target of PA effect [44, 45]. In addition to macrophages, neutrophils also produce NO [46]. Therefore, the reduction in neutrophil recruitment into the peritoneal cavity might have accounted for the PA reduction of carrageenan-induced NO production in the peritoneal cavity. NO has a significant role as a microbicidal molecule. However, NO reacts with superoxide anion forming peroxynitrite, which presents deleterious effects such as tissue lesion [4]. Therefore, the effect of PA of limiting superoxide anion and NO production may have as a consequence the decrease of tissue lesions together with the reduction of oxidative stress. Evidence demonstrates that PA induces vasorelaxation in an eNOS-derived NO production manner [47]. However, in the present experimental condition, the NO production is dependent on iNOS and not eNOS, which is in agreement with *in vitro* data demonstrating that PA inhibits LPS-induced iNOS mRNA and protein expression by macrophages [12].

Cytokines are important mediators of inflammation. In fact, targeting cytokines reduces carrageenan-induced paw edema, leukocyte recruitment and myeloperoxidase activity [25, 48]. PA inhibited carrageenan-induced TNF-α and IL-1β production in the peritoneal exudates. The cytokines selection took into account their proven role in inflammatory diseases and as therapeutic targets [49]. PA has been shown to inhibit cytokine production via inhibition of NFκB. For instance, PA inhibits LPS-induced IL-6 production by RAW 264.7 macrophages by inhibiting the mitogen-activated protein kinases p38 and ERK (extracellular-regulated kinase) [12]. *In vivo*, PA inhibited carrageenan-induced hyperalgesia and NFκB activation in the paw skin [14]. The inhibition of NFκB activation might also explain the reduction of NO production since the expression of iNOS is NFκB dependent [50]. Therefore, it is reasonable that the PA anti-inflammatory effect observed in the present study might depend on inhibiting carrageenan-induced NFκB activation.

It is noteworthy to mention that despite PA inhibition of carrageenan-induced leukocyte recruitment, superoxide anion, and NO production, it is unlikely that PA treatment would facilitate infection since PA is an antimicrobial molecule [51]. PA inhibits the growth of endodontic bacteria such as *Porphyromonas gingivalis*, *Prevotella nigrescens*, *Prevotella intermedia*, *Prevotella buccae*, *Fusobacterium nucleatum*, *Bacteroides fragilis*, *Actinomyces naeslundii*, *A*. *viscosus*, *Peptostreptococcus micros*, *Enterococcus faecalis and Aggregatibacter actinomycetemcomitans* [51], bacteria involved in caries such as *Streptococcus salivarius*, *S*. *sobrinus*, *S*. *mutans*, *S*. *mitis*, *S*. *sanguinis and Lactobacillus casei* [52], and gram positive bacteria such as *Bacillus cereus*, *B*. *subtilis*, *Enterococcus faecalis*, *E*. *hirae*, *Kocuria rhizophila*, *Staphylococcus aureus* strains, *S*. *epidermidis*, *Streptococcus agalactiae*, *S*. *dysgalactiae*, *S*. *pneumonia*, *S*. *pyogenes* [53].

Concluding, the present study demonstrates the anti-inflammatory effect of PA on carrageenan-induce leukocyte recruitment and edema and suggests some possible mechanisms of action such as inhibition of oxidative stress, cellular activation, NO and cytokine production.
